# Discovery of *Hemilepistus elongatus* Budde-Lund, 1885 (Isopoda, Oniscidea) in Iran: redescription and intraspecific character variability

**DOI:** 10.3897/zookeys.176.2271

**Published:** 2012-03-20

**Authors:** Ghasem M. Kashani, Alireza Sari

**Affiliations:** 1 Department of Biology, Faculty of Science, University of Zanjan, Zanjan, Iran; 2 School of Biology, College of Science, University of Tehran, Tehran, Iran

**Keywords:** Oniscidea, *Hemilepistus elongatus*, redescription, character variability

## Abstract

In the present study, *Hemilepistus elongatus* Budde-Lund, 1885 is reported from Iran for the first time, redescribed and its diagnostic characters are figured. This species reveals a high variability in morphological characters. The division of the species at the subspecific level can not be supported anymore. This species differs from other species of the genus by the unique shape of male pleopod-endopodite I.

## Introduction

Budde-Lund (1879) created *Hemilepistus* as a subgenus of the genus *Porcellio* Latreille, 1804. Verhoeff (1930) raised it to the generic level and divided it into two subgenera, *Hemilepistus* and *Desertellio*, based on absence or presence of a frontal line between the profrons and the vertex. Originating in Central Asia (Schmalfuss 1998), this genus has expanded its geographical distribution to North Africa (Lincoln 1970). Recently, Kashani et al. (2010) reported five species of the subgenus *Hemilepistus* from Iran and this is the first record of the subgenus *Desertellio* from this region. According to the species list of Schmalfuss (2003), the subgenus *Desertellio* includes 10 valid species, namely *Hemilepistus buddelundi* Borutzky, 1945, *Hemilepistus communis* Borutzky, 1945, *Hemilepistus elongatus* Budde-Lund, 1885, *Hemilepistus fedtschenkoi* (Uljanin, 1875), *Hemilepistus heptneri* Borutzky, 1945, *Hemilepistus nodosus* Budde-Lund, 1885, *Hemilepistus pavlovskii* Borutzky, 1954, *Hemilepistus ruderalis* (Pallas, 1771), *Hemilepistus russonovae* Borutzky, 1951 and *Hemilepistus zachvatkini* Verhoeff, 1930.

*Hemilepistus elongatus* was described by Budde-Lund (1885) on the basis of one female specimen from Taschburun in “Transcaucasus”. Borutzky (1945) reported this species from Caucasus and later he (Borutzky 1955) described the new subspecies *Hemilepistus elongatus transcaspius* from Turkmenistan. In addition to the above mentioned localities, Röder et al. (1993, 1996) and Röder and Linsenmayr (1999) reported this species from Ararat, easternmost Turkey. No record of this species has been reported from Iran. The present study, however, showed that this species has a broad geographical distribution in Iran.

**Figure 1.**
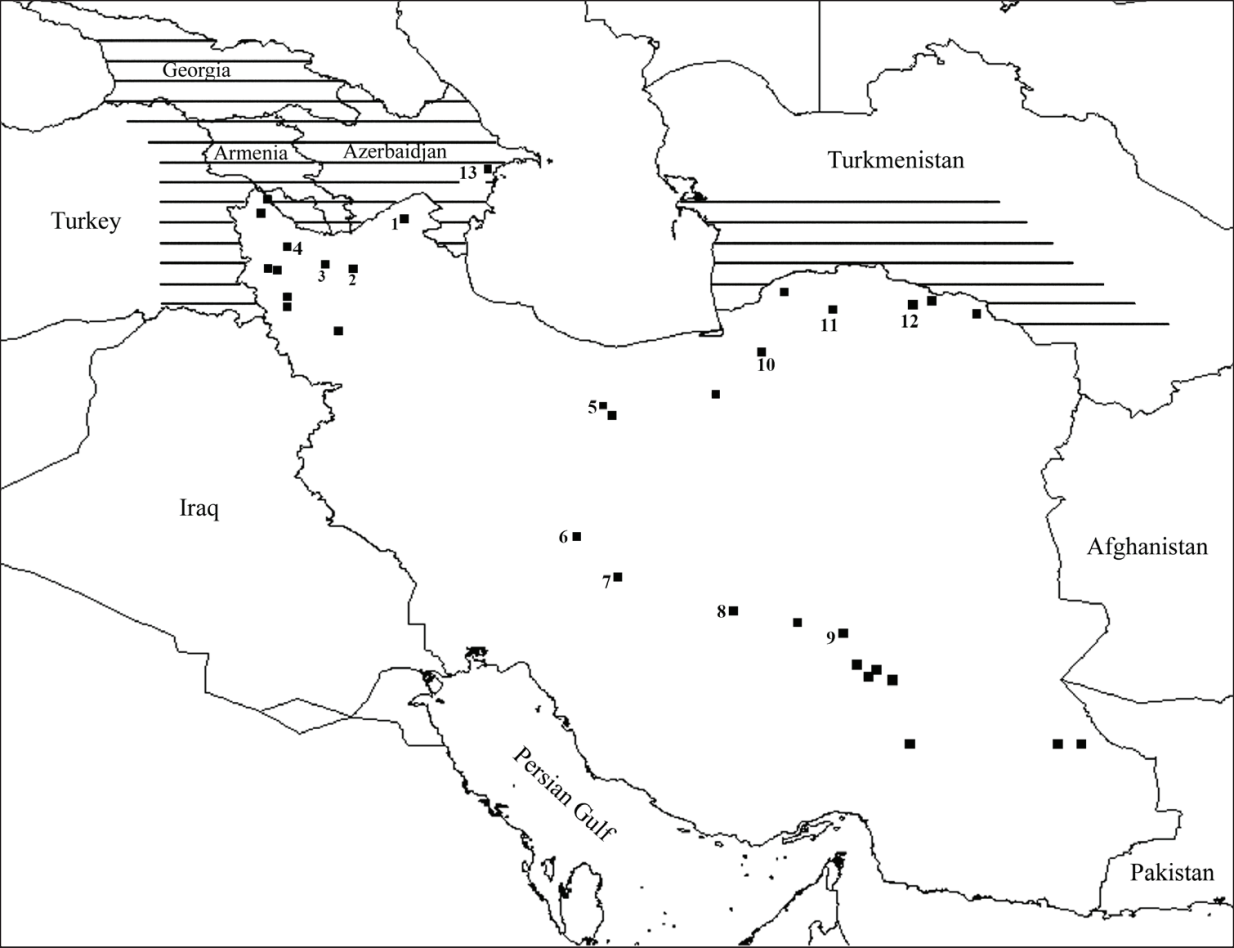
Sampling localities of *Hemilepistus elongatus* from Iran along with its distribution area in other countries (striped line). Numbers refer to the localities in the subsequent figures.

Intraspecific variability of morphological characters has been reported in many terrestrial isopods (e.g. in *Oniscus asellus* (Bilton 1994), *Ligidium* spp. (Klossa-Kilia et al. 2006), *Porcellio lamellatus* (Montesanto et al. 2007), *Orthometopon* spp. (Poulakakis and Sfenthourakis 2008), *Armadillo tuberculatus* (Kamilari and Sfenthourakis 2009)). Examination of numerous specimens from different parts of Iran (Fig. 1) and some other specimens from Caucasus revealed that intraspecific variability is present also in some diagnostic characters of *Hemilepistus elongatus*.

Due to the lack of a comprehensive description along with a critical consideration of subspecific division for the species, the main purposes of the present paper are the redescription of *Hemilepistus elongatus*, the demonstration of its character variability and the elucidation of its taxonomic status at subspecific level. Moreover, new records of this species from Iran are presented.

## Material and methods

The material of the present study from Iran was collected by the first author unless otherwise mentioned. The specimens were collected by hand and preserved in 96% ethanol. The isopods were dissected and body parts were slide-mounted using Euparal (Carl Roth, Karlsruhe). Digital color images were taken using a Qimaging MicroPublisher 5.0 RTV digital camera and Syncroscopy Auto-Montage (v 5.03.0061) software. Drawings were made using a camera lucida fitted on an Olympus SZX12 dissecting stereomicroscope and on an Olympus BX51 compound microscope. The material used for SEM-preparations was air-dried overnight. The mounted material was coated with gold in a sputter coater to 40 nm thickness and examined with a Hitachi S-2460N SEM.

For comparison, type or additional material was obtained from the Natural History Museum, London (BMNH) and Staatliches Museum für Naturkunde, Stuttgart (SMNS). The examined material from Iran is deposited in the Zoological Museum, University of Tehran (ZUTC). Some specimens are kept in the personal collection of the first author.

## Results and discussion

In addition to the presence of *Hemilepistus elongatus* in Caucasus, Turkey and Turkmenistan, the present study confirms the occurrence of this species from Iran where it has a broad geographical distribution (Fig. 1) in diverse habitats. This species, like many other terrestrial isopods, shows a high variability in many morphological characters including coloration, size and shape of frons, ratio of flagellar articles, shape of pleotelson, and even in the male secondary sexual structures such as pereiopod VII ischium and pleopod I (see description). This character variability led Borutzky (1955) to propose a new subspecies, *Hemilepistus elongatus transcaspius*, for specimens from Turkmenistan. According to the Russian author, this subspecies differed from the typical *Hemilepistus elongatus* in the shape of the pleopod-endopodite and exopodite I. Evaluation of numerous specimens from different localities in Iran and Caucasus revealed that various states of these characters are sometimes found within the same population, often with intermediate forms. Though there was no possibility to examine specimens from Turkmenistan, the differences which led Borutzky (1955) to describe a new subspecies, fall within the morphological variability of *Hemilepistus elongatus*.

### Family Agnaridae Schmidt, 2003

#### Hemilepistus elongatus

Budde-Lund, 1885

http://species-id.net/wiki/Hemilepistus_elongatus

Hemilepistus elongatus Budde-Lund, 1885: 160.– Walter 1889: 1110.– Röder et al. 1996: 818.– Ziegler and Miller 1997:181.– Röder and Linsenmayr 1998: 57.– 1999: 349.– Jeppesen 2000:238.
Hemilepistus (Desertellio) elongatus Borutzky 1945:198.
Hemilepistus (Desertellio) elongatus transcaspius Borutzky 1955: 217.– 1961: 26
Desertellio elongatus Röder et al., 1993: 339.

##### Material examined.

**Iran:** Marand to Ghare-Ziaoddin, 38˚35.5’N, 45˚15.7’E, 8 November 2004, leg. A. Kazemi, one male and one female (ZUTC Iso.1080); Bilesavar to Parsabad, 39°33.8'N, 47°56.7'E, 16 June 2008, one male and one female (ZUTC Iso.1081); Tabriz to Khaje, 38°08.7'N, 46°35.7'E, 17 June 2008, one male and one female (ZUTC Iso.1082); Tabriz to Marand, 38°14.9'N, 47°06.6'E, 18 June 2008, one female (ZUTC Iso.1083); Poldasht to Makoo, 39°17.0'N, 44°42.7'E, 18 June 2008, one female (ZUTC Iso.1084); Urmia, Golmankhaneh port, 37°35.7'N, 45°15.3'E, 2 October 2008, one male and two females (ZUTC Iso.1085); Shahin-Dezh to Miandoab, 36°52.5'N, 46°17.3'E, 3 October 2008, two males and two females (ZUTC Iso.1086); Tabriz, Agh-Gonbad port, 17 June 2008, one female (ZUTC Iso.1087); Shirvan, 37°25.1'N, 57°52.7'E, 6 May 2008, one male (ZUTC Iso.1088); Abadeh to Semirom, 31°05.8'N, 51°56.3'E, 9 April 2008, one male and one female (ZUTC Iso.1089); Varamin, Pishva, 35°12.2'N, 51°48.2'E, 23 June 2008, one female (ZUTC Iso.1090); Zahedan to Khash, 28°32.8'N, 60°49.4'E, 28 February 2009, one female (ZUTC Iso.1091).

##### Additional material.

**Turkey:** Holotype, female, Caucasus, Taschburun, in A. Brandt collection, leg.?, det. Budde-Lund (BMNH 1921.10.18–4103); **Turkmenistan:** Tschikischljar, 27 April 1986, leg.?, one female (BMNH 1921.10.18–4102); **Azerbaidjan:** S. Baku, 20 km N Salyani, 30 May 1996, leg. W. Schawaller, det. H. Schmalfuss, one male and one female (SMNS 11530); **Georgia:** Caucasus, Vashlovan Reserve, 7–9 May 1983, leg. Golovatch, det. H. Schmalfuss, one male and one female (SMNS 13082); **Iran:** 150 km N Isfahan, 4 June 1975, leg. Bauer, det. H. Schmalfuss, one female (SMNS 11020).

##### Diagnosis.

Cephalothorax with rounded lateral lobes and short to developed median lobe, frons with or without incision in the middle; dorsal part with several rounded tubercles of the same or different size. Pereion-tergites I to III with tubercles decreasing in number posteriorly. Male pereiopod VII ischium with straight to sinuate ventral margin. Male pleopod-endopdite I straight; apex with a leaf-like lobe.

##### Redescription.

**Figure 2.**
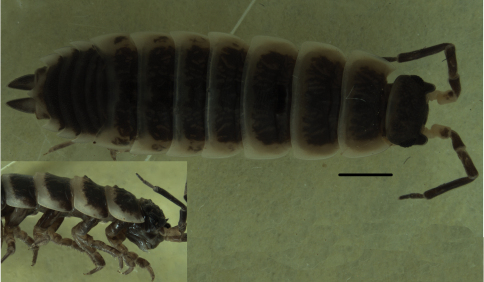
*Hemilepistus elongatus* from Isfahan, Semirom to Abadeh (7). Female, dorsal view and lateral view of head and first four pereionites. Scale, 2 mm.

Maximum length of both male and female: 18 mm. Body elongated. Color brown with epimera, posterior margin of tergites and pleotelson pale (Fig. 2).

**Figure 3.**
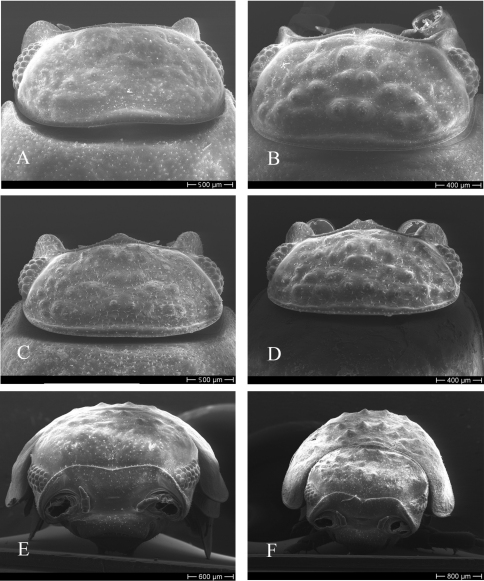
*Hemilepistus elongatus*. Cephalothorax; **A–D** dorsal view; **E–F** frontal view **A** from Kerman, Bardsir (9), 12 mm long **B** from Khorasan, Shirvan (12), 12 mm long **C** from Alborz, Karaj (5), 13 mm long **D** from Tabriz, Soofian (3), 11 mm long **E** from Kerman, Bardsir (9), 14 mm long; **F** from Tabriz, Marand (4), 14 mm long.

**Figure 4.**
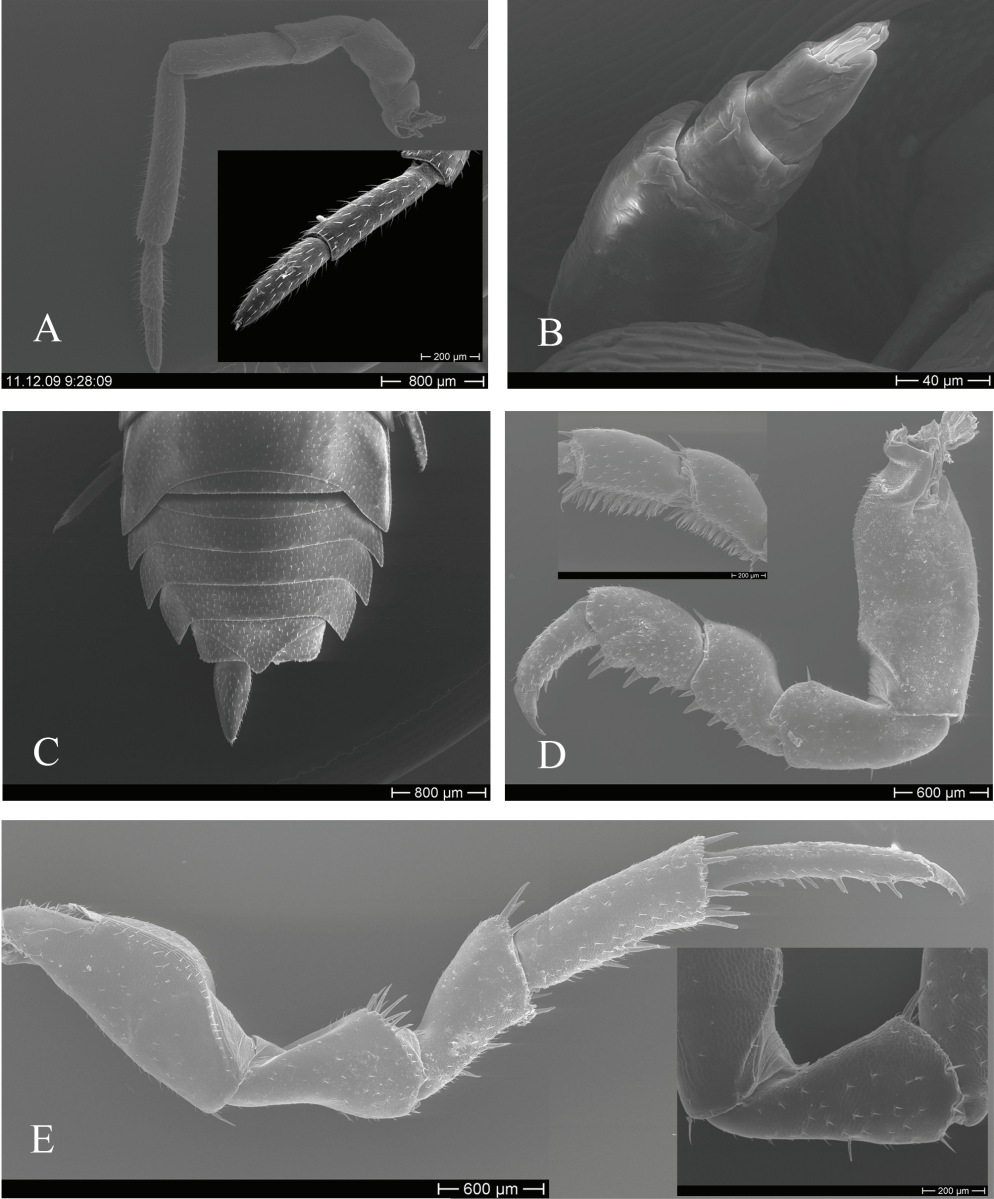
*Hemilepistus elongatus*. **A** antenna **B** antennule **C** pleon **D** male pereiopod I from Khorasan, Shirvan (12) and Ardabil, Parsabad (1) (merus & carpus) **E** male pereiopod VII from Ardabil, Parsabad (1) and Tabriz, Soofian (3) (ischium).

Cephalothorax with rounded lateral lobes, median lobe with variable size and shape; several rounded tubercles of the same or different size in dorsal part (Fig. 3A–D); frontal line sinuous in frontal view, with or without incision in the middle; no suprantennal line (Fig. 3E–F); eyes with 20–25 ommatidia. Antenna long, reaching posterior margin of second pereion-tergite; flagellum slightly shorter than fifth article of peduncle, with two articles, first article of equal length or up to 2.5 times as long as second article (Fig. 4A). Antennule of three articles with a tuft of long aesthetascs at apex (Fig. 4B).

**Figure 5.**
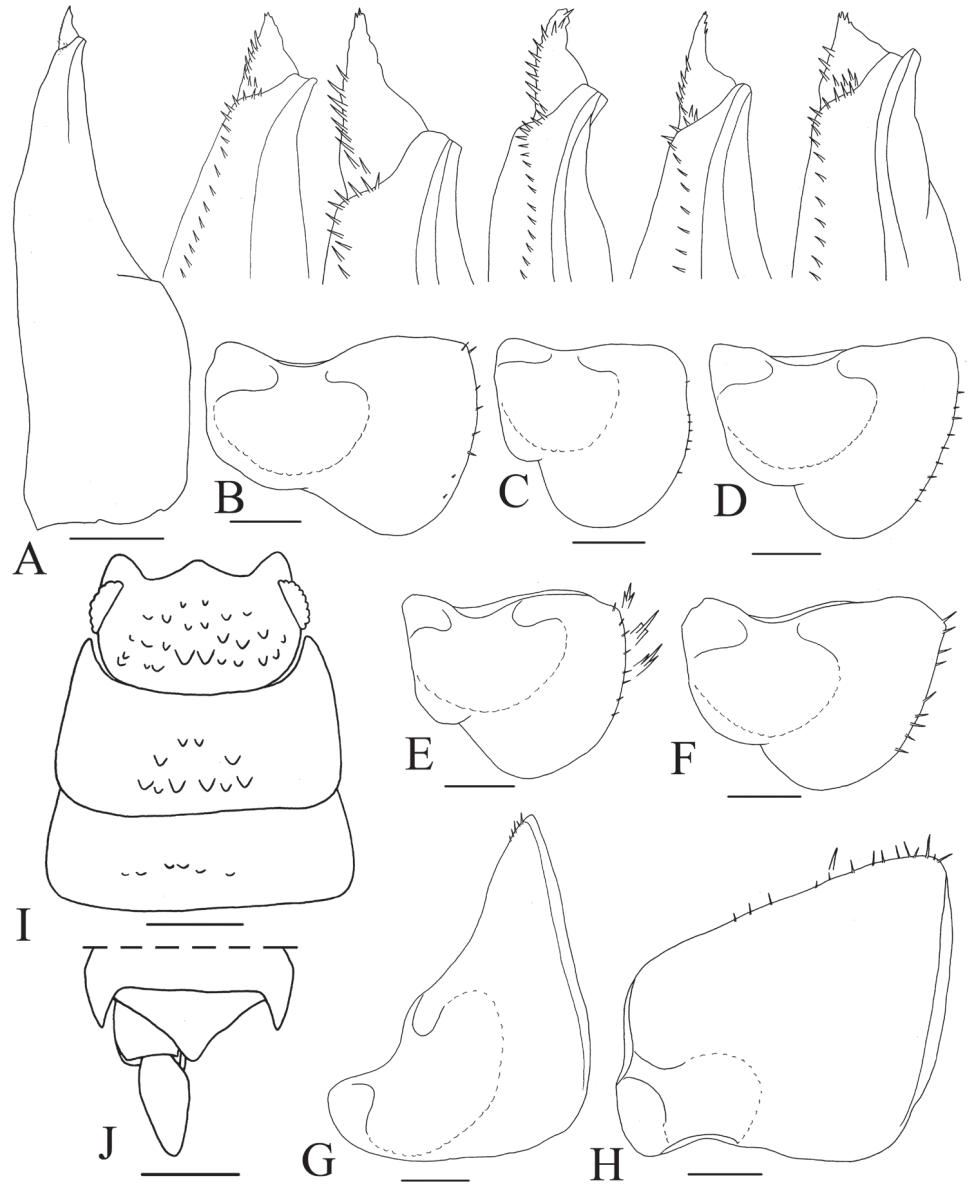
*Hemilepistus elongatus*. **A** male pleopod-endopodite I and five enlarged apex, left to right from Azerbaijan, S Baku (13), Isfahan, Tiran (6), Fars, Saadatshahr (8), Semnan, Kalate-Khij (10) and Ardabil, Parsabad (1) **B–F** male pleopod-exopodite I **B** from Northen Khorasan, Ashkhaneh to Minoo-Dasht (11) **C** from Ardabil, Parsabad (1) **D** from Tabriz, Khajeh (2) **E** from Azerbaijan, S Baku (13) **F** from Isfahan, Semirom to Abadeh (7) **G** male pleopod-exopodite II, from Azerbaijan, S Baku (13) **H** male pleopod-exopodite III, from Azerbaijan, S Baku (13) **I** **holotype**, cephalotorax and pereion-tergites I-II **J holotype**, pleotelson. Scales, 0.5 mm for **A–H** and 1 mm for **I-J**.

Pereion-tergite I with rounded tubercles, two markedly larger tubercles on the median part; and with rounded hind margin (Fig. 5I). Pereion-tergites II–III with fewer tubercles. Pereion-tergites IV-VII smooth.

Pleon short, smooth, slightly narrower than pereion (Fig. 4C). Pleotelson short, triangular, with slightly concave sides and acute, rounded or truncate apex slightly surpassing uropod-protopodites (Fig. 5J). Uropod-exopodites conical, about twice as long as protopodites

Pleopod-exopodites I–V with monospiracular covered lungs.

Male: Pereiopod I merus and carpus with or without brushes of setae (Fig. 4D). Pereiopod II to VII with no brushes of setae on merus and carpus.Pereiopod VII ischium with straight or sinuate ventral margin; merus and carpus equipped with strong setae (Fig. 4E).

Pleopod-endopodite I straight, apex with a leaf-like lobe, equipped with setae, variable in shape (Fig. 5A). Pleopod-exopodite I as in Fig. 5B–F, inner lobe variable in shape. Pleopod-exopodites II-III as in Fig. 5G–H.

##### Remarks.

This species is distinguished from other species of the genus by the unique shape of male pleopod-endopodite I, with apex bearing a leaf-like lobe.

##### Distribution.

Georgia; Azerbaijan; easternmost Turkey; Turkmenistan; Iran.

## Supplementary Material

XML Treatment for Hemilepistus elongatus
